# Effect of methionine hydroxy analog feed supplements: Significant alteration and enrichment of rumen microbiota and metabolome in Hu sheep

**DOI:** 10.3389/fvets.2022.999726

**Published:** 2022-10-26

**Authors:** Shujie Li, Hanfang Zeng, Changjian Wang, Zhaoyu Han

**Affiliations:** College of Animal Science and Technology, Nanjing Agricultural University, Nanjing, China

**Keywords:** methionine hydroxy analog, rumen fermentation, rumen microbiota, rumen metabolome, Hu sheep

## Abstract

Methionine hydroxy analogs (MHA) are widely used as the main sources of methionine in ruminant feed production. The purpose of this study was to explore the effect of using MHA supplements such as MHA as a salt of calcium (MHA-Ca) and 2-hydroxy-4-(methylthio)-butanoic acid isopropyl ester (HMBi) as sources of methionine on the rumen microbiota and metabolome in Hu sheep. Seventy-two healthy Hu sheep were randomly assigned to three dietary treatment groups: control, MHA-Ca, and HMBi groups. The results showed that the concentrations of total volatile fatty acids, acetate, and propionate were higher in the HMBi group than in the control group. The HMBi and MHA-Ca groups had higher alpha diversity values than those in control group. We compared the rumen microbiota by using 16S rRNA gene sequencing. At the phylum level, the HMBi group had a higher relative abundance of Firmicutes and a lower relative abundance of Synergistetes than did the control group. At the genus level, the control group had a higher relative abundance of *Treponema_2* than did the HBMi group and a higher relative abundance of *Prevotellaceae_UCG_004* than did the MHA-Ca group. Metabolomic analyses revealed that fatty acids, amino acids, lipids, organic acids, sugars, amines, and nucleosides were significantly altered in both MHA-Ca and HMBi groups. Metabolites with significant differences were enriched in amino acid and carbohydrate metabolisms, such as phenylalanine metabolism, biosynthesis of amino acids, tryptophan metabolism, galactose metabolism, and tyrosine metabolism. Above all, the findings presented in this study indicate that MHA alter the rumen microbiota and metabolites and that different forms of MHA have different impacts. The results of our study contribute to a better understanding of the effects of MHA.

## Introduction

Many studies have reported that methionine (Met) is the first or second limiting amino acid in ruminant protein synthesis because of its low concentration in dietary feed (1). Met is degraded by microorganisms after entering the rumen, which results in a lesser quantity of Met reaching the small intestine for utilization and absorption. One approach for the protection of Met from degradation in the rumen is to supply methionine hydroxy analogs (MHA) that resist microbial breakdown (2). The isopropyl ester of 2-hydroxy 4-(methylthio)-butanoic acid (HMBi) and calcium salt of the hydroxy analog of methionine (MHA-Ca) are two different forms of MHA. HMBi is produced by the esterification of 2-hydroxy-4-methylthiobutyric acid with isopropanol (3). MHA-Ca is obtained by neutralizing an MHA with calcium hydroxide or calcium oxide, which is then dried, crushed, and sieved (4). It has been determined that ~50% of MHA is directly absorbed by the rumen wall and converted into Met and the rest is metabolized in the rumen utilized by the rumen microorganisms of adult cattle (5, 6). Previous studies have shown that supplementation with HMBi results in increased milk fat content and protein yield (7, 8). Extensive research has also indicated that that dietary supplementation of HMBi can improve the growth performance and the feed efficiency of finishing beef cattle by potentially changing bacterial community (6). Evidence suggests that the addition of MHA-Ca to methionine-deficient diets can significantly improve nitrogen deposition in pigs (9). The addition of 0.15% MHA-Ca to the diet of lactating dairy cow can effectively improve the production performance, milk quality, and feed digestion as well as utilization (10). Studies on the effects of MHA on rumen fermentation have shown conflicting results. MHA has no apparent effect on rumen fermentation (11). However, dietary supplementation with 2-hydroxy-4-(methylthio)-butanoic acid (HMB) and HMBi increased the concentrations of volatile fatty acids (VFA) and the abundance of *Lactobacillus* and *Flavobacterium* in the rumen (12).

The rumen is a complex microbial ecosystem inhabited by various microorganisms such as bacteria, protozoa, fungi, and viruses (13). According to previous research, dietary changes can have a significant effect on the microbiota (14). The gut microbiota can have dramatic effects on host development and metabolism. Studies in mice suggest that gut microbiota may influence brain development and function (15). Changes in metabolite production may also affect the fatty acid metabolism of hosts, thereby altering their lifespan (16). MHA is partially degraded in the rumen and can serve as a source of Met for rumen microorganisms. Whether MHA can affect animal growth and development by altering rumen microbiota and metabolism remains unknown. This lack of knowledge has resulted in an inability to properly evaluate the effects of MHA on ruminant feed production.

Hu sheep are native to China and are excellent for both wool and meat (17). Previous research have mainly concentrate on the effects of MHA on the performance of high-production dairy cows (18, 19). Few studies have investigated the effect of MHA on rumen microbiota and metabolome in Hu sheep. Consequently, the main purpose of this research was to describe the effects of consuming feed with different MHA on the rumen microbiota and metabolome in Hu sheep. In addition, the relationship between the rumen microbiota and metabolome was also determined.

## Materials and methods

### Animals, diets, and experimental design

Seventy-two healthy Hu sheep comprised half male and half female (60–70 days old and weighing 19.99 ± 2.04 kg) were randomly divided into three dietary treatment groups: control (CON), MHA-Ca, and HMBi groups. Each group consisted of 24 Hu sheep, half male and half female, divided into 6 replicates (each replicate consisted of 4 sheep of the same sex), and each replicate was housed in individual pen. The sheep house was a semi-closed sheep house. The experimental period was autumn in China. The temperature range was 5–25°C, the relative humidity was 40–80%, and the light was natural light. All the sheep were fed twice a day at 06:00 h and 19:00 h, and water was available over the course of the experiment. All animals were fed a pelleted total mixed ration diet. The diet was formulated in conformity with the feeding standards of meat-producing sheep (NY/T816-2004, China). The nutrient composition of the basic diet was essentially the same among the three treatments except for the supplementation of MHA. The chemical compositions and ingredients of the diets are listed in Table 1. The CON group was fed the same basal diet with no rumen-protected Met, whereas diets of the MHA-Ca and HMBi groups were additionally supplemented with 1.5 kg/t MHA-Ca and 1.5 kg/t HMBi. There were 80 days in the experimental period, with the first 7 days being used for preadaptation.

**Table 1:** Composition and nutrient levels of the basal diet (DM basis) %.

**Ingredients**	**Content, % of DM**	**Chemical composition, % of DM^4^**	**Content, % of DM**
Corn	30.8	DM	87.19
Soybean meal	5.8	ME/(MJ/kg)	16.91
Peanut meal	3.0	CP	14.61
Bean straw	24.0	NDF	49.71
Corn germ meal	15.0	ADF	13.04
Rice husk	5.0	EE	4.71
Wheat middling	2.0	Ash	10.06
Molasses	1.0	Ca	1.08
Malt root	6.0	P	0.62
NH_4_Cl	0.4		
Limestone	2.0		
Premix	5.0		
Total	100		

The premix provided the following per kg of the diet: VA 16000 IU, VD 25000 IU, VE400 IU, Fe 35 mg, Cu 20 mg, Zn 65 mg, Mn 40 mg, I 0.2 mg, Se 0.3 mg, Co 0.15 mg, MHA 1.5 g.

### Sample collection and measurements

All Hu sheep were kept food-deprived for 24 h and water-deprived for 2 h. Then, one sheep from each replicate was randomly selected for slaughter (i.e., a total of six sheep from each group). We collected the ruminal fluid samples immediately after slaughter and strained them through cheesecloth. Approximately 20 mL of ruminal fluid was stored in liquid nitrogen for the determination of fermentation parameters, extraction of microbial DNA, and metabolome measurements. The fermentation parameters were analyzed using gas chromatography (7890A, Agilent, Palo Alto, CA, USA) according to the procedure by Mao et al. (20).

### Microbial DNA extraction and sequencing

Using the DNA extraction kit (QIAGEN, D Neasy Power Soil Kit 10) to extract the total genomic DNA of the ruminal fluid sample. The purity and concentration of the DNA was verified with Nano Drop and agarose gel. Taking an appropriate amount of the sample into a centrifuge tube, dilute the sample with sterile water to 1 ng/μ L. Using the diluted genomic DNA as the template, according to the selection of the sequencing region, using the specific primers with Barcode, Takara Ex Taq high-fidelity enzyme from Takara Company for PCR to ensure the amplification efficiency and accuracy. For bacterial diversity analysis, V3-V4 (or V4-V5) variable regions of 16S rRNA genes was amplified with primer pairs 343F (5'- TACGGRAGGCAGCAG−3') and 798R (5'- AGGGTATCTAATCCT-3').

### Rumen microbiota bioinformatic analyses

Raw data is in FASTQ format. The original paired-end sequences were descrambled using Trimmomatic version 0.35 software (21). Detected and truncated the ambiguous base N and used the sliding window method to check the average base quality (21). When the quality was lower than 20, the preceding high-quality sequence was truncated. The paired-end sequence after decontamination was performed using FLASH version 1.2.11 software (22). The splicing parameters were as follows: the smallest overlap was 10 bp, the largest overlap was 200 bp, and the largest mismatch rate was 20%. To ensure the accuracy of the results, precise impurity removal could be performed to remove sequences containing ambiguous bases, homologous single bases, and sequences that were too short in length. The parameters for precise impurity removal were: remove sequences containing N bases, and retain sequences with a base quality score Q20 of at least 75% (23). After the sequencing data was preprocessed to generate high-quality sequences, the Vsearch version 2.4.2 software was used to classify the sequences into multiple OTUs according to the similarity of the sequences (24). The parameter was sequence similarity greater than or equal to 97% to be classified as an OTU unit (25). The representative sequences of each OTU were selected using the QIIME version 1.8.0 software (http://qiime.org/scripts/index.html) and all representative sequences were aligned and annotated with the database (26). 16S was aligned with Green genes or Silva version123 database, species alignment was annotated with RDP classifier software, and annotation results with confidence intervals >0.7 were retained (24). The relative abundance of the phylogenetic investigation of communities by reconstruction of unobserved states PICRUSt - predicted metabolic pathways of ruminal bacterial microbiome in three groups. The extended error bar plot was generated using STAMP software (http://kiwi.cs.dal.ca/Software/).

### Metabolomic measurement

Eighty microliter of sample was transfered into 1.5 ml EP tubes. Then 10 μL of internal standard (L-2-chlorophenylalanine, 0.3 mg/mL in methanol) was added and vortexed for 10 s. Two hundred forty microliter of methanol-acetonitrile (2:1) mixed solution were added and vortexed for 1 min. Ultrasonic extraction in ice water bathed for 5 min and were stand at−20°C for 10 min. The sample was centrifuged for 10 min (12,000 rpm, 4°C) and transfered 150 μL of the supernatant into a glass derivatization bottle. Quality control samples (QCs) were prepared by mixing equal volumes of extracts from all samples and each QC had the same volume as the sample. Eighty microliter of methoxyamine hydrochloride in pyridine (15 mg/mL) was added to a glass derivatized vial, vortexed for 2 min and performed oximation in a shaking incubator at 37°C for 90 min. After the sample was taken out, 80 μL of BSTFA (containing 1% TMCS) derivatization reagent and 20 μL of n-hexane were added. The sample was left at room temperature for 30 min for GC-MS metabolomic analysis.

The samples were tested on an Agilent 7890B gas chromatography system and an Agilent 5977A MSD system (Agilent Technologies Inc., CA, USA). The derivatives were separated to utilized the DB-5MS fused-silica capillary column (30 m × 0.25 mm × 0.25 μm, Agilent J & W Scientific, Folsom, CA, USA). The injection volume was 1 μL and the injection was splitless with a solvent delay of 5 min. The temperature of the injection port was 260°C.The initial temperature of the column oven was 60°C, the temperature was programmed to 125°C at 8°C/min, and 5°C/min was heated to 210°C; 10°C/min was heated to 270°C, 20°C/min to 305°C and held for 5 min.

### Rumen microbiota data preprocessing and statistical analysis

The data matrix was imported into SIMCA software (version 14.0, Umetrics, Umea, Sweden) and autonomous principal component analysis (PCA) was used to determine the overall distribution among samples and the process of general analysis. The partial least squares analysis (PLS-DA) and orthogonal partial least squares analysis (OPLS-DA) were used to distinguish the overall differences in metabolic profiles between groups and to find differential metabolites between groups. The combination of multidimensional analysis and single-dimensional analysis were used to compare the differential metabolites between groups. In the OPLS- DA analysis, the variable weight value (Variable important in projection, VIP) was used to measure the impact strength and explanatory power of the expression pattern of each metabolite on the classification and discrimination of each group of samples. Metabolites with VIP>1 were considered differential metabolites. Further *t*-test (student's *t*-test) was used to verify whether the metabolite differences between groups were significant.

### Data analyses

The data on ruminal fermentation parameters were analyzed using a one-way analysis of variance (SPSS v. 25.0, SPSS Inc., Chicago, IL, USA). The results were expressed as mean ± standard deviation, and *p* < 0.05 was considered a significant difference. Principal coordinate analysis was used to detect differences between the microbial communities from different experimental groups. Wilcoxon-Mann-Whitney *U*-test was used to identify phylum and genus-level differences in microbes. Metabolites with variable influence on projection (VIP) values larger than 1.0 and *p*-values from a two-tailed Student's *t*-test < 0.05 were considered differential metabolites. The correlation matrix between bacterial families and altered metabolite levels was generated using Spearman's correlation coefficients and visualized using R language.

## Results

### Ruminal fermentation parameters

The HMBi group had higher concentrations of total volatile fatty acid, acetate, and propionate (*p* = 0.03, *p* = 0.02, and *p* = 0.03, respectively), than the CON group. However, there was no significant difference in pH, butyrate, isovalerate, and valerate concentrations between the three groups (Table 2).

**Table 2:** Ruminal fermentation parameters of CON, MHA-Ca and HMBi groups.

**Item**	**CON**	**MHA-Ca**	**HMBi**	***P-*value**
pH	6.99 ± 0.03	7.02 ± 0.02	7.01 ± 0.03	0.33
Acetate (mmol/L)	31.97 ± 4.34^b^	47.57 ± 7.95^ab^	55.98 ± 4.96^a^	0.02
Propionate (mmol/L)	8.16 ± 1.10^b^	12.71 ± 2.52^ab^	16.21 ± 2.60^a^	0.03
Isobutyrate (mmol/L)	1.71 ± 0.35	2.45 ± 0.38	2.36 ± 0.54	0.34
Butyrate (mmol/L)	4.83 ± 0.52	8.21 ± 1.98	8.68 ± 1.01	0.08
Isovalerate (mmol/L)	2.83 ± 0.56	3.72 ± 0.76	3.83 ± 0.97	0.41
Valerate (mmol/L)	0.72 ± 0.08	1.23 ± 0.28	1.31 ± 0.29	0.14
Total VFA (mmol/L)	50.24 ± 6.39^b^	75.92 ± 13.42^ab^	88.39 ± 9.49^a^	0.03
A:P	3.96 ± 0.25	4.03 ± 0.41	3.69 ± 0.41	0.63
Acetate (%)	63.65 ± 2.08	64.08 ± 2.81	64.06 ± 2.53	0.91
Propionate (%)	16.20 ± 0.61	16.19 ± 0.81	18.13 ± 1.81	0.31
Butyrate (%)	9.71 ± 0.23	10.12 ± 1.19	9.78 ± 0.44	0.95

CON (Control group) = sheep fed total mixed ration pellet diet with a basal diet. MHA-Ca (MHA-Ca group) = sheep fed total mixed ration pellet diet additionally supplemented with MHA-Ca. HMBi (HMBi group) = sheep fed total mixed ration pellet diet additionally supplemented with HBMi. VFA = volatile fatty acid. A: P = the ratio of acetate to propionate. In the same row, values with different small letter superscripts mean significant difference (P < 0.05) while with the same letter or no letter superscripts mean no significant difference (P>0.05). The same as below.

### Richness, diversity, and composition of the ruminal bacterial communities

**Figure 1:**
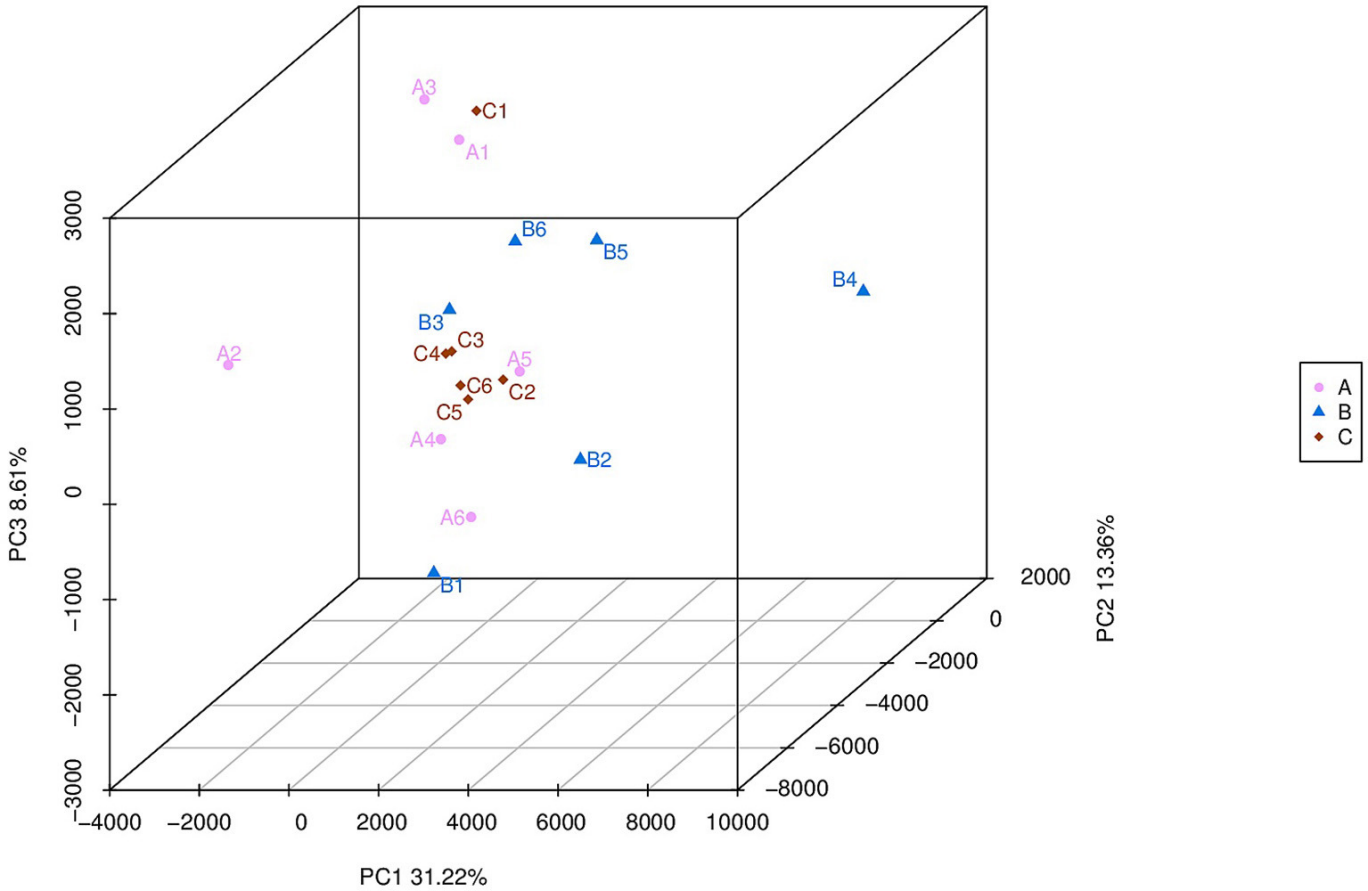
A=CON group,B=MHA-Ca group,C=HMBi group; Unweighted UniFrac metric PCoA of microbial diversity in CON group, MHA-Ca and HMBi groups. The percentage of variation explained by PC1, PC2, PC3 are indicated on the axis.

The number of observed species was higher (*p* = 0.015) in the HMBi group than in the CON group. The Chao 1 value was higher (*p* < 0.01) in both MHA-Ca and HMBi groups than that in the CON group (Table 3). There was no significant difference in the Shannon–Wiener and Simpson indices among the three groups. According to the principal coordinate analysis profile (Figure 1), the CON, MHA-Ca, and HMBi groups were detached (axis 1 + axis 2 + axis 3 = 55.2%).

**Table 3:** Alpha diversity of ruminal bacterial communities of CON, MHA-Ca and HMBi groups.

**Item**	**CON**	**MHA-Ca**	**HMBi**	***P-*value**
Observed species	1493.08 ± 75.88^b^	1682.88 ± 41.13^ab^	1818.88 ± 81.80^a^	0.015
Chao 1 value	1795.61 ± 105.59^b^	2073.17 ± 36.09^a^	2195.98 ± 77.29^a^	0.008
Shannon wiener	7.44 ± 0.11	7.29 ± 0.23	7.85 ± 0.21	0.125
Simpson index	0.98 ± 0.01	0.96 ± 0.01	0.98 ± 0.01	0.282

CON (Control group) = sheep fed total mixed ration pellet diet with a basal diet. MHA-Ca (MHA-Ca group) = sheep fed total mixed ration pellet diet additionally supplemented with MHA-Ca. HMBi (HMBi group) = sheep fed total mixed ration pellet diet additionally supplemented.

a, b The same letters indicate no significant difference in functional abundance between the two groups; different letters indicate significant differences in functional abundance between the two groups.

In total, we identified 18 bacterial phyla in the ruminal fluid samples. Abundant taxa (relative abundance ≥ 0.01%) are presented in Table 4. The analyzed DNA sequences typically belonged to the phyla Bacteroidetes, Firmicutes, and Proteobacteria. We found that the HMBi group had a significantly higher (*p* = 0.04) relative abundance of Firmicutes than the CON group did. In contrast, the relative abundance of Synergistetes was significantly higher in the CON group than that in the HMBi group. No significance was found in the abundance of Bacteroidetes, Spirochaetae, Tenericutes and other bacterial phyla between the three groups.

**Table 4:** The relative abundance of phylum level (% of total sequences) in ruminal bacterial communities of CON, MHA-Ca and HMBi group.

**Phylum**	**Relative abundance (%)**			***P*-Value**
	**CON**	**MHA-Ca**	**HMBi**	
*Bacteroidetes*	72.73 ± 2.41	70.14 ± 4.91	73.51 ± 1.49	0.75
*Firmicutes*	12.90 ± 1.45^b^	14.43 ± 0.63^ab^	16.11 ± 1.22^a^	0.04
*Proteobacteria*	7.35 ± 1.18	10.15 ± 5.25	5.10 ± 1.16	0.23
*Spirochaetae*	2.63 ± 0.31	1.92 ± 0.26	1.68 ± 0.41	0.14
*Fibrobacteres*	2.01 ± 0.39	1.27 ± 0.26	1.77 ± 0.27	0.27
*Tenericutes*	1.78 ± 0.48	1.38 ± 0.34	1.15 ± 0.27	0.51
*Lentisphaerae*	0.24 ± 0.07	0.27 ± 0.03	0.19 ± 0.06	0.62
*Other*	0.11 ± 0.03	0.15 ± 0.03	0.19 ± 0.02	0.23
*Elusimicrobia*	0.08 ± 0.05	0.12 ± 0.05	0.10 ± 0.03	0.83
*Actinobacteria*	0.07 ± 0.01	0.04 ± 0.01	0.06 ± 0.03	0.47
*Cyanobacteria*	0.02 ± 0.01	0.03 ± 0.01	0.04 ± 0.01	0.48
*Saccharibacteria*	0.02 ± 0.006	0.02 ± 0.005	0.03 ± 0.006	0.48
*Synergistetes*	0.024 ± 0.006^a^	0.011 ± 0.004^ab^	< 0.01^b^	0.04

CON (Control group) = sheep fed total mixed ration pellet diet with a basal diet. MHA-Ca (MHA-Ca group) = sheep fed total mixed ration pellet diet additionally supplemented with MHA-Ca. HMBi (HMBi group) = sheep fed total mixed ration pellet diet additionally supplemented with HBMi.

a, b The same letters indicate no significant difference in functional abundance between the two groups; different letters indicate significant differences in functional abundance between the two groups.

In total, we identified 190 taxa in the ruminal fluid samples. The abundant taxa (relative abundance ≥ 0.01%) are presented for clarity and visualization in Table 5. The relative abundance of *Ambiguous taxa* was higher (*p* = 0.01) in both the MHA-Ca and HMBi groups than in the CON group. In the CON group, the relative abundance of *Prevotellaceae_UCG_004* was higher (*p* = 0.04) than that in the MHA-Ca group, whereas the relative abundance of *Ruminococcaceae_UCG_014 taxa* was lower (*p* = 0.03) than that in the HBMi group. In addition, a significant increase (*p* = 0.04) in the relative abundance of *Treponema_2* was observed in the CON group compared with that in the HBMi group. The other genera showed no significant difference between the CON, MHA-Ca, and HMBi groups.

**Table 5:** The relative abundance of Genus level (% of total sequences) in ruminal bacterial communities of CON, MHA-Ca and HMBi group.

**Genus**	**Relative abundance (%)**			***P*-value**
	**CON**	**MHA-Ca**	**HMBi**	
*Prevotella_1*	29.67 ± 3.69	26.21 ± 2.26	28.23 ± 4.51	0.79
Other	12.34 ± 0.79	11.57 ± 1.60	12.55 ± 1.58	0.87
uncultured_rumen_bacterium	10.17 ± 1.28	9.01 ± 1.89	9.92 ± 0.83	0.82
*Rikenellaceae_RC9_gut_*group	6.75 ± 0.87	9.66 ± 1.34	8.74 ± 1.23	0.23
*Ambiguous_taxa*	2.03 ± 0.81^b^	5.58 ± 0.95^a^	4.97 ± 0.37^a^	0.01
*Prevotellaceae_UCG_001*	4.79 ± 0.42	3.23 ± 0.44	3.66 ± 0.66	0.12
*Succinivibrio*	2.01 ± 0.78	8.65 ± 4.34	1.01 ± 0.34	0.10
*Prevotellaceae_UCG_003*	3.67 ± 0.99	3.26 ± 0.61	3.26 ± 0.71	0.91
uncultured	2.57 ± 0.42	3.63 ± 0.83	3.63 ± 1.13	0.60
*Erysipelotrichaceae_UCG_004*	3.34 ± 1.04	1.55 ± 0.17	1.62 ± 0.29	0.11
*Treponema_2*	2.47 ± 0.29^a^	1.69 ± 0.26^ab^	1.47 ± 0.35^b^	0.04
*Fibrobacter*	2.01 ± 0.39	1.26 ± 0.26	1.74 ± 0.26	0.27
*Ruminococcaceae_UCG_002*	0.84 ± 0.32	1.21 ± 0.31	1.24 ± 0.47	0.70
*Prevotellaceae_UCG_004*	1.37 ± 0.44^a^	0.50 ± 0.11^b^	1.25 ± 0.25^a^	0.03
*uncultured_bacterium*	1.25 ± 0.40	0.84 ± 0.37	0.94 ± 0.34	0.71
*Lachnospiraceae_ND3007_*group	0.95 ± 0.28	1.10 ± 0.19	0.91 ± 0.13	0.79
*Alloprevotella*	0.87 ± 0.26	0.82 ± 0.34	0.85 ± 0.27	0.99
*Thalassospira*	0.98 ± 0.31	0.51 ± 0.18	1.01 ± 0.69	0.67
*Anaerovibrio*	1.06 ± 0.47	0.54 ± 0.09	0.33 ± 0.05	0.19
*Ruminococcaceae_UCG_*014	0.44 ± 0.12^b^	0.41 ± 0.07^b^	1.04 ± 0.28^a^	0.04
*Ruminococcaceae_UCG_*010	0.58 ± 0.15	0.64 ± 0.11	0.59 ± 0.19	0.95
*Selenomonas_1*	0.61 ± 0.18	0.64 ± 0.31	0.38 ± 0.11	0.66
*Ruminococcus_1*	0.74 ± 0.15	0.45 ± 0.12	0.40 ± 0.10	0.15

CON (Control group) = sheep fed total mixed ration pellet diet with a basal diet. MHA-Ca (MHA-Ca group) = sheep fed total mixed ration pellet diet additionally supplemented with MHA-Ca. HMBi (HMBi group) = sheep fed total mixed ration pellet diet additionally supplemented with HMBi.

a, b The same letters indicate no significant difference in functional abundance between the two groups; different letters indicate significant differences in functional abundance between the two groups.

### Predicted functions of ruminal bacterial microbiota

**Figure 2:**
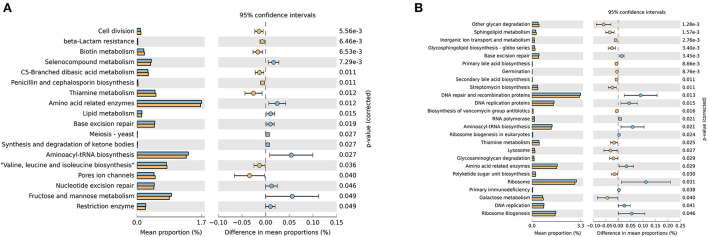
The relative abundance of the phylogenetic investigation of communities by reconstruction of unobserved states (PICRUSt) - predicted metabolic pathways of ruminal bacterial microbiome in three groups: the CON group vs. MHA-Ca group **(A)**; the CON group vs. HMBi group **(B)**. The extended error bar plot was generated using STAMP software. Welch's two-sided test was used, and Welch's inverted test was 0.95.

This study inferred that 18 gene families in the ruminal microbiota showed significantly different abundances between the CON and the MHA-Ca groups (Figure 2A), and 25 gene families in the ruminal microbiota showed significantly different abundances between the CON and HMBi groups (Figure 2B). Furthermore, compared with the CON group, the gene families involved in base excision repair, aminoacy-tRNA biosynthesis, and amino acid-related enzymes were significantly decreased, whereas that for thiamine metabolism was significantly increased in both the MHA-Ca and HMBi groups.

### GC/MS analysis of the ruminal fluid

**Figure 3:**
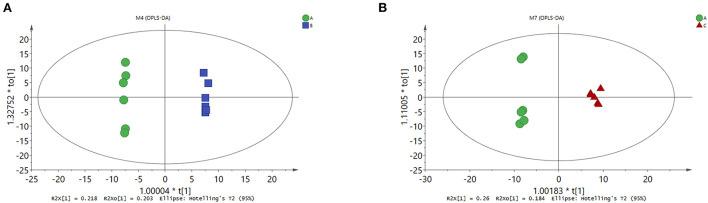
OPLS-DA score plots derived from the GC/MS metabolite profiles of rumen samples. OPLS-DA score plots (respectively) for: the CON group vs. MHA-Ca group **(A)**; the CON group vs. HMBi group **(B)**.

To explore whether changes in rumen microbes could lead to alterations in rumen metabolites, GC/MS-based metabolome profiling was used to characterize rumen metabolism. A total of 238 metabolites were found in the rumen samples, including amino acids, organic acids, lipids, sugars, amines, and nucleosides. Orthogonal partial least squares discriminant analysis was performed on the metabolites in the CON, MHA-Ca, and HMBi groups. The score plot showed a clear separation between the CON and MHA-Ca groups (Figure 3A) and between the CON and HMBi groups (Figure 3B). This indicates the differences in metabolites in the rumen among the three groups.

### Differences in ruminal metabolites between CON, MHA-Ca, and HMBi groups

In addition, we compared these three sets of data to detect differences in metabolites between the three groups. In total, 62 differential metabolites were detected between MHA-Ca and CON, and 84 differential metabolites were observed between the HMBi and CON groups. Based on the statistical analysis and the VIP value obtained from the partial least squares discriminant analysis (false discovery rate < 0.05, and VIP > 1), 9 differential metabolites were identified in MHA-Ca and CON groups, 24 differential metabolites were identified in the HMBi and CON groups. These metabolites were shown in Tables 6, 7, respectively. Generally, the ruminal metabolites differed mainly in fatty acids, amino acids, organic acids, amines, and nucleosides among the three groups.

**Table 6:** List of ruminal fluid metabolites that showed significant difference between MHA-Ca and CON groups.

**Metabolite name**	**Average RT**	**Quant mass**	**VIP**	**FDR**	**log2(FC)**
Stigmasterol	30.21	144	1.969	< 0.001	−1.988
Hydrocinnamic acid	13.52	104	1.910	0.003	−0.693
Arabitol	19.92	217	1.867	0.005	−0.697
Kynurenine	8.87	192	1.827	0.005	−1.049
Quinic acid	22.73	345	1.817	0.023	−0.788
2,4,7-Trihydroxypteridine	33.79	381	1.839	0.027	−0.817
5-Methoxypsoralen	6.53	173	1.801	0.027	−0.403
Chlorogenic acid	34.02	307	1.751	0.027	−0.811
Ribulose-5-Phosphate	25.59	317	1.676	0.027	−0.702

RT = retention time. VIP = variable importance in projection. FDR = false discovery rate. log2(FC) = The ratio of the average expression of metabolites in the two groups of samples, positive value means up-regulation, negative value means down-regulation.

**Table 7:** List of ruminal fluid metabolites that showed significant difference between HBMi and CON groups.

**Metabolite name**	**Average RT**	**Quant mass**	**VIP**	**FDR**	**log2(FC)**
Resveratrol	32.18	445	1.856	< 0.001	2.261
Hydrocinnamic acid	13.52	104	1.820	< 0.001	−0.613
Kynurenine	8.87	192	1.861	< 0.001	−1.075
Arabitol	19.92	217	1.785	< 0.001	−0.662
4-Hydroxycinnamic acid	25.62	293	1.910	< 0.001	1.008
N-Acetylornithine	21.54	174	1.853	0.001	−0.918
Sorbitol	20.03	221	1.841	0.001	−0.656
4-(5-Methyl-2-Furanyl)-2-Butanone	6.49	152	1.829	0.001	−0.487
Epicatechin	30.77	179	1.676	0.001	2.299
N-Acetylputrescine	15.3	174	1.639	0.005	1.350
Hexaric acid	26.25	333	1.669	0.006	−0.477
Uridine 5'-Monophosphate	21.44	312	1.733	0.007	1.117
Nicotianamine	17.03	348	1.546	0.009	1.471
Tyrosol	31.3	179	1.550	0.011	1.630
3-Hydroxybutyric acid	8.45	191	1.624	0.012	0.710
2-Hydroxybutanoic acid	6.14	207	1.552	0.017	0.309
Adenosine	32.75	230	1.771	0.022	2.652
2-Monostearin	34.84	411	1.543	0.022	−1.294
Ethanolamine	5.27	174	1.618	0.024	−0.366
Pipecolic acid	11.23	156	1.630	0.031	−1.247
Cholesterol	36.32	329	1.413	0.045	−0.965
4-Aminobutyric acid	15.88	174	1.603	0.045	−0.636
N-Methylglutamic Acid	10.29	98	1.122	0.045	−1.421
5-Hydroxy-3-Indoleacetic acid	28.75	337	1.636	0.047	−1.825

RT = retention time. VIP = variable importance in projection. FDR = false discovery rate. log2(FC) = The ratio of the average expression of metabolites in the two groups of samples, positive value means up-regulation, negative value means down-regulation.

### Metabolic pathways of differential metabolites

**Figure 4:**
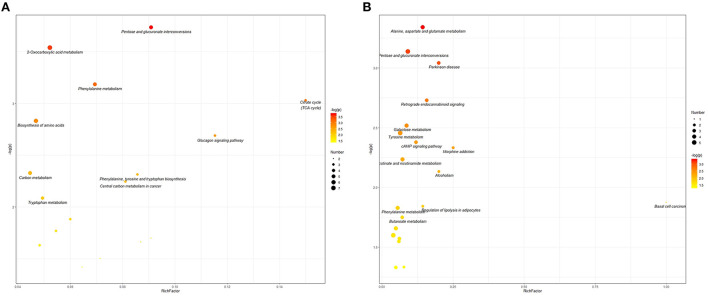
Metabolome view map of the differential metabolites identified in three groups: the CON group vs. MHA-Ca group **(A)**; the CON group vs. HMBi group **(B)**. The larger size indicates higher pathway enrichment, and darker color indicates higher pathway impact values.

Pathway analysis was conducted to supply a inclusive view of the different metabolites between the Hu sheep in the CON, MHA-Ca, and HMBi groups. Results revealed that pentose and glucuronate interconversions, 2-oxocarboxylic acid metabolism, citrate cycle, biosynthesis of amino acids, glucagon signaling pathway, carbon metabolism, phenylalanine, tyrosine, and tryptophan biosynthesis, central carbon metabolism in cancer, phenylalanine metabolism, and tryptophan metabolism were the top 10 pathways which were significantly enriched in MHA-Ca group compared with CON group (Figure 4A). Results of the comparison between the HMBi and CON groups showed that alanine, aspartate, and glutamate metabolism, Parkinson's disease, retrograde endocannabinoid signaling, galactose metabolism, tyrosine metabolism, cAMP signaling pathway, pentose and glucuronate interconversions, morphine addiction, nicotinate and nicotinamide metabolism, and alcoholism were the top 10 pathways which were significantly enriched in HMBi group compared with CON group (Figure 4B).

### Correlation analyses between the ruminal metabolome and microbiome

**Figure 5:**
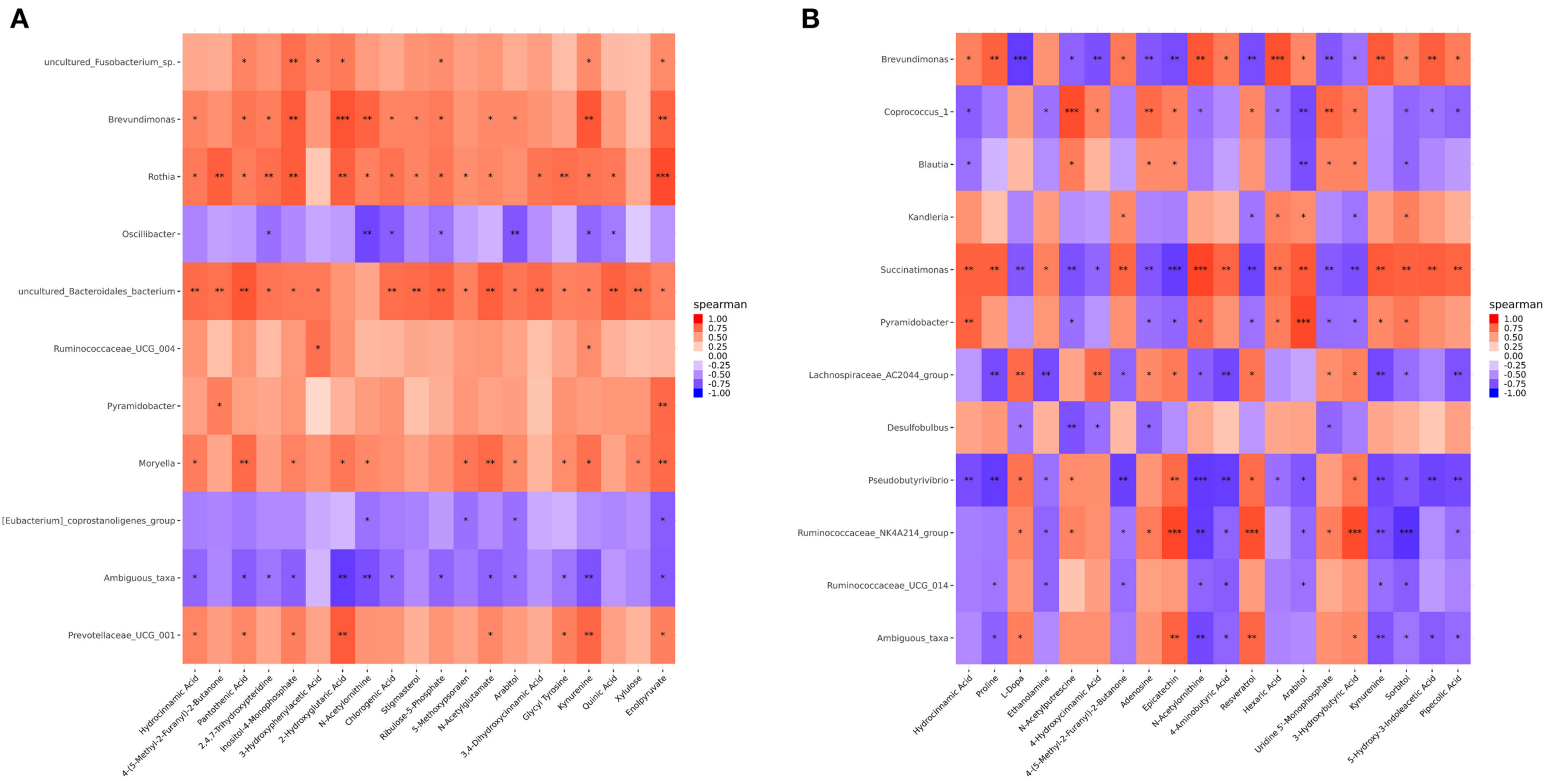
Correlation analyses between the differential metabolites and top 20 ruminal bacterial genera in three groups: the CON group vs. MHA-Ca group **(A)**; the CON group vs. HMBi group **(B)**. The blue squares represent the positive correlations while orange ones represent the negative correlations. *p < 0.05, **p < 0.01; ***p < 0.001.

Correlation analyses were performed using Spearman's correlation coefficients obtained for the microorganisms and differential metabolites in the CON, MHA-Ca, and HMBi groups to investigate potential host-microbiota metabolic interactions. The results of these analyses indicate a complex connection between rumen microbiota and metabolites. For example, the Spearman's correlation constructed using data from the CON and MHA-Ca groups showed that *Oscillibacter, Ambiguous taxa*, and *[Eubacterium]_coprostanoligenes_group* were negatively correlated with most metabolites (Figure 5A), and in the CON and HMBi groups, *Ruminococcaceae_UCG_014* was negatively associated with eight metabolites (including proline, sorbitol, 4-aminobutyric acid, and kynurenine) (Figure 5B).

## Discussion

For Hu sheep, the rumen plays an important role in digestion, metabolism, and health (27). VFA, such as acetate, butyrate, and propionate, are the end products of rumen microbial fermentation and are considered one of the rumen fermentation indexes (28). Dietary or feed additives can affect ruminal fermentation patterns. Changes in rumen VFA concentrations may reflect changes in rumen fermentation patterns (29, 30). In our study, the results showed that MHA can significantly enhance the rumen fermentation characteristics of Hu sheep, and the concentrations of acetate, butyrate, and total VFA were higher in both the MHA-Ca and HMBi groups than in the CON group. These results are consistent with those of Zhou (31), who also found that HMBi can increase the concentrations of acetate, butyrate, and total VFA *in vitro*. Previous studies have reported that MHA can promote rumen fermentation, acetate concentration was significantly decreased by HMBi deduction (6, 32). A possible explanation for this might be that MHA could use free ammonia nitrogen in the rumen to synthesize Met, thereby increasing the amount of Met in the rumen. Met promotes the establishment of microflora and improves the digestibility of nitrogenous compounds and carbohydrates in the rumen.

As an indicator of the ruminal microbiota physiological state, the microbiota's richness and diversity are important. Ruminal bacterial microbiota can be influenced by dietary feed (33). In the present study, we investigate the impact of MHA feeding on the composition and diversity of the rumen bacterial community. At the phylum level, Firmicutes and Bacteroidetes were the two major phyla in all three groups. At the genus level, *Prevotella_1, uncultured rumen bacterium, Rikenellaceae_RC9_gut_group*, and *Ambiguous taxa* were highly abundant. In addition, The HMBi and MHA-Ca groups had higher alpha diversity values than those in control group, which showed that the microbial community composition was changed and tended to be more diverse in Hu sheep in both the HMBi and MHA-Ca groups. These results are consistent with those of previous research (34, 35), and previous studies have found that Met deficiency hinders the growth and reproduction of rumen bacteria and protozoa (36). MHA promotes rumen bacterial growth (37), and MHA and HMBi supplementation increase VFA concentrations in the rumen and the ruminal abundance of *Fibrobacter succinogenes* and *Ruminococcus flavefaciens* (12). This result can be explained by the fact that MHA can be used by ruminal bacterial microbiota to promote the proliferation of rumen microorganisms. Another important finding was that the MHA-Ca and HMBi groups had a higher relative abundance of Firmicutes than did the CON group. Firmicutes are a core bacterial component of the rumen (38). It contains many fiber-decomposing bacteria, including *Ruminococcus, Butyvibrio, and Eubacterium*. The main function of Firmicutes is to degrade fiber and cellulose (39, 40). Previous research have revealed that dandelions (as potential functional feed additives) could improve the abundance of rumen Firmicutes bacteria, and a higher abundance of Firmicutes could enhance rumen fermentation (41). When the supply of HMBi was reduced, the ruminal microbiota was inhibited, which led to a decrease in the Shannon index and the relative abundance of Firmicutes (32). This is consistent with our study in which the concentrations of total VFA, acetate, and propionate were higher in both the MHA-Ca and HMBi groups than in the CON group. A possible explanation for this might be that MHA-Ca and HMBi can be metabolized to MHA in the rumen and thus enhance rumen fermentation by increasing the abundance of rumen Firmicutes bacteria.

Metabolomics explains phenotypic changes better than genomics or proteomics (42). To better understand the effects of MHA on rumen microorganisms, we conducted a metabolomic study. Our data showed that the concentrations of many rumen metabolites changed in both the MHA-Ca and HMBi groups, which might be associated with changes in rumen microbial abundance. Compared with the CON group, several organic acids, such as hydrocinnamic, quinic, chlorogenic, 4-hydroxycinnamic, and pipecolic acids, were less abundant in both the MHA-Ca and HMBi groups. In contrast, levels of 4-hydroxycinnamic, 3-hydroxybutyric, and 2-hydroxybutanoic acids were higher in the HMBi group than in the CON group. These organic acids, among other functions, are involved in host development and metabolism. Several reports have shown that hydrocinnamic acids exhibit antioxidant potential (43). Quinic acid, an organic acid precursor of aromatic amino acids, is integral to the metabolism of shikimic acid (44, 45). Chlorogenic acid is a polyphenol with a strong bacteriostatic activity (46, 47). Kynurenine, arabitol, sorbitol, and N-acetylornithine were also identified. In addition, correlation analysis revealed that arabitol and sorbitol had a high positive correlation with *Pyramidobacter* and *Succinatimonas*. N-acetylornithine and kynurenine were negatively correlated with *Ambiguous taxa* and *Ruminococcaceae_UCG_014*. Using the Kyoto encyclopedia of genes and genomes analysis, we found that the metabolites with significant differences were mainly enriched in amino acid and carbohydrate metabolisms, such as phenylalanine metabolism, tryptophan metabolism, alanine, aspartate, and glutamate metabolism, biosynthesis of amino acids, galactose metabolism, and tyrosine metabolism. This is consistent with earlier observations, which showed that the contents of microbial amino acids such as phenylalanine, methionine, isoleucine, and leucine in the rumen liquid and solid phases were significantly increased when fed coated Met and adsorbed MHA (48). Previous research have identified that the metabolism of the rumen microbiota is affected by substrate amino acids (49). Met was used as a nitrogen and carbon source to study its effect on amino acid metabolism of rumen microorganisms, and it was found that adding Met can improve the utilization of valine, serine, cysteine, and histidine, change the metabolism of histidine, and increase the content of the product (50). These results indicate that MHA has a significant effect on amino acid and carbohydrate metabolisms.

## Conclusion

In summary, the present study was designed to determine the effect feeding an MHA to Hu sheep had on the rumen microbiota and metabolome. These findings indicate that ruminal fermentation was stimulated, as evidenced by increased concentrations of total VFA, acetate, and propionate. The composition of the bacterial community, such as *Firmicutes* and *Synergistetes*, was altered, and the richness and diversity of the ruminal microbiota were significantly enhanced with MHA supplementation. Metabolomic analysis revealed that some ruminal metabolites were significantly altered, including amino acids, organic acids, and amines. Moreover, correlation analysis of the metabolome and microbiome showed some associations between the microbial groups and metabolites. In general, this study contributes to our understanding of the use of MHA as a feed supplement in Hu sheep.

## Data availability statement

The datasets presented in this study can be found in online repositories. The names of the repository/repositories and accession number(s) can be found at: https://www.ncbi.nlm.nih.gov/, PRJNA833900.

## Ethics statement

The animal study was reviewed and approved by Administration of Affairs Concerning Experimental Animals (The State Science and Technology Commission of P. R. China, 1988. No. SYXK (Su) 2015-0656).

## Author contributions

SL and ZH designed the experiments. SL, HZ, and CW conducted the experiments. SL and HZ performed the analysis of the experimental data. Finally, the manuscript was written by SL and ZH. All authors have read and agreed to the published version of final manuscript.

## Funding

This study was financially supported by Technical System of Modern Agriculture (Dairy cow) Industry in Jiangsu Province JATS [2022] 469.

## Conflict of interest

The authors declare that the research was conducted in the absence of any commercial or financial relationships that could be construed as a potential conflict of interest.

## Publisher's note

All claims expressed in this article are solely those of the authors and do not necessarily represent those of their affiliated organizations, or those of the publisher, the editors and the reviewers. Any product that may be evaluated in this article, or claim that may be made by its manufacturer, is not guaranteed or endorsed by the publisher.
